# Synthesis and Pharmacological Evaluation of Schiff Bases of 4-(2-Aminophenyl)-Morpholines

**DOI:** 10.4103/0250-474X.57292

**Published:** 2009

**Authors:** P. Panneerselvam, M. Gnanarupa Priya, N. Ramesh Kumar, G. Saravanan

**Affiliations:** Department of Pharmaceutical Chemistry, C. L. Baid Mehta College of Pharmacy, Jyothi Nagar, Old Mahabaliupuram Road, Thoraipakkam, Chennai-600 096, India

**Keywords:** Morpholine, analgesic, antiinflammatory, antibacterial, antifungal

## Abstract

In the present study, a novel series of 4-(2-aminophenyl)morpholines were synthesized and characterized by IR, ^1^H-NMR, ^13^C NMR and mass spectral analysis. The synthesized compounds were screened for analgesic (100 and 200 mg/kg), antiinflammatory (200 and 400 mg/kg), antibacterial (*Bacillus subtilis, Bacillus cereus, Staphylococcus epidermidis, Staphylococcus aureus, Klebsiella pneumoniae, Pseudomonas aeruginosa* and *Escherichia coli*) and antifungal (*Candida albicans* and *Aspergillus niger*) activities. The minimum inhibitory concentrations of the compounds were also ascertained by agar streak dilution method. N-benzylidine-2-morpholoino benzenamine (1) and N-(3-nitro benzylidine)-2-morpholino benzenamine (3) exhibited significant analgesic, antiinflammatory and antimicrobial activities.

4-Phenyl morpholine derivatives have been reported to possess antiinflammatory[1–3] antimicrobial[4,5] and central nervous system activities[6,7]. Schiff bases have been reported to possess biological properties[8,9] apart from antimicrobial activities[10–13]. In our previous work[14], it was observed that substitution of 4-phenyl morpholine to quinazoline moiety results in potent analgesic, antiinflammatory, and antimicrobial activities. Linezolide also possess a 4-pheny1 morpholine substitution. Moreover morpholine as a substitution in many heterocyclic moieties has been reported to possess analgesic, antiinflammatory[14], central nervous system and antimicrobial activities[15]. In addition, in general nitro/chloro/furan substituted compounds have been reported to possess various biological activities.

In continuation of our earlier work[14,15] on 2-methyl quiuazolin-4(3H)-ones we attempted to synthesize the new series of Schiff bases of 4-(2-aminophenyl)-morpholines and its characterization by IR, NMR and mass spectral analysis. The synthesized compounds were screened for analgesic activity by chemical writhing and Eddy's hot plate method, antiinflammatory activity by paw edema method, anti-bacterial and anti-fungal activities. The minimum inhibitory concentrations of the compounds were also ascertained by agar dilution method.

Morpholine and 1-chloro-2-nitrobenzene was refluxed to yield 4-(2-nitrophenyl) morpholine, which was then reduced using zinc and hydrochloric acid affords 4-(2-aminophenyl) morpholine[16]. An equimolor mixture of 1.78 g (0.01 mol) of 4-(2-aminopheny1) morpholine and substituted aldehydes (aromatic/heterocyclic) in absolute ethanol was refluxed using dean stark apparatus for 2 h (Scheme 1). The reaction mixture was cooled for a while and solid separated was filtered and recrystallised from ethanol (99%) yield the title compounds (1-5). The structural assignment of the products was based on their IR,^1^H-NMR, ^13^C-NMR and mass spectral data.

**Scheme 1:**
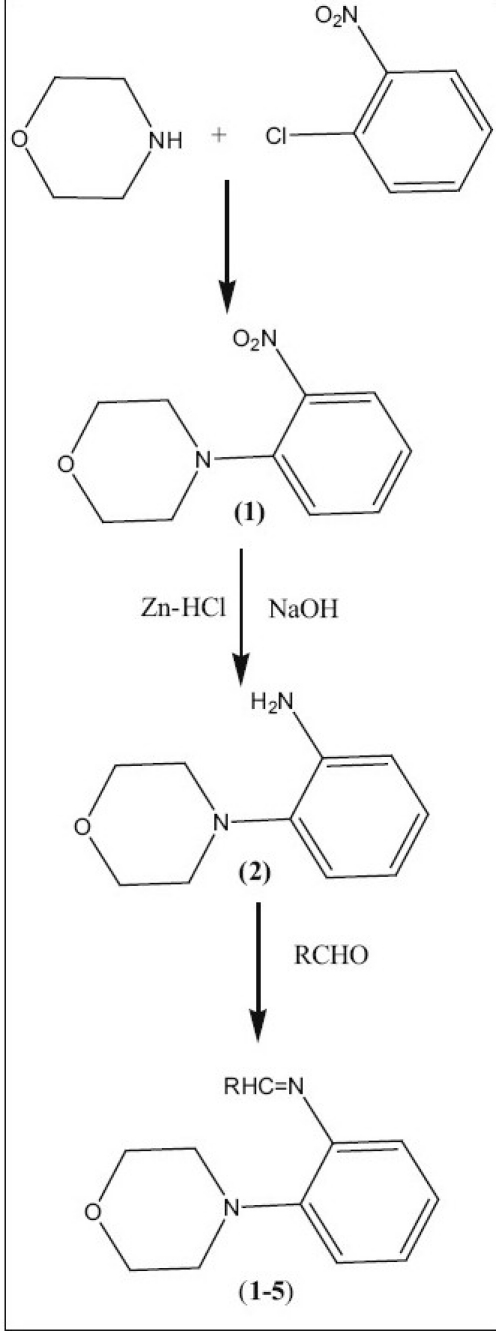
Synthetic route for the preparation of 4-(2-aminophenyl)-morpholines 1: R = C_6_H_5_, 2: R = furyl, 3: R = m-NO_2_C_6_H_4_, 4: R = o-ClC_6_H_5_ and 5: R = -CH=CHC_6_H_5_

IR (KBr) cm^−1^: 2950 (Ar-H), 2852 (N=CH), 1630 (C=N), 1503 (C=C), 1261 (C-N), 1114 (C-O-C), 848 (Ar-H), 1580 (N-O), 710 (C-Cl). ^1^H-NMR (CDCI_3_) δ: 8.12 (s, 1H: N=CH), 7.82-7.73 (m, 2H; 2”, 6”-H), 7.48-7.46 (m, 3H; 3”, 4”, 5”-H); 7.28-7.30 (d; J=8.9 Hz, 2H; 3',5'-H), 6.97-6.99 (d, J=9.1 Hz, 2H; 4',6'-H), 6.95-6.96 (d, J=6 Hz, 1H; CH=CH Ph), 3.89-3.92 (t, J=8.1 Hz' 4H, 2, 6-CH_2_), 3.23-3.25 (t, J=7.6 Hz, 4H; 3,5-CH_2_). ^13^C-NMR (CDC13) δ: 154.3, 139.7, 136.7, 131.3, 129.0, 128.2, 127.6, 127.3, 116.0, 66.9, 50.3. MS (E1) m/z: 266 (M^+^).

Melting points were determined in open capillary tubes and are uncorrected. IR spectra were recorded (in KBr) on an ABB Bomen MB-104. NMR spectra were recorded on 300 MHz-Bruker DPX200 using tetramethylsilane as internal standard. Mass spectra were recorded on a Shimadzu GC-MS QP5000. The purity of the synthesized compounds were checked by TLC using E-Merck TLC aluminum sheets silica gel 60F 254 (0.2 mm) using ethyl acetate:hexane (2:3) as eluent and visualized in an iodine chamber. All the chemicals used were of analytical grade. Unpaired student-t-test[17] was performed to ascertain the significance of the exhibited analgesic and antiinflammatory activities.

Swiss mice (15-25 g) and Wistar rats (150-200 g) were used for these studies[18]. They were kept in colony cages at 25±2^°^, relative humidity of 45-55% under 12 h light and dark cycles. All the animals were acclimatized for a week before use. The animals were fed with standard animal feed and water *ad libitum.* The test compounds were administered orally using intragastric tube in the form of suspension using 5% Tween 80 as suspending agent. The experimental does was selected between the minimum effective dose and maximal non-lethal dose. All the animal experimentations were performed according to the protocols and recommendations of the Institutional Animal Ethics Committee. Unpaired student t-test was performed to ascertain the significance of the exhibited analgesic and antiinflammatory activities.

The analgesic activity[19] was determined by acetic acid-induced writhing method using Swiss mice (n=6) of either sex selected by random sampling technique was used for the study. Aspirin at a dose level of 100 mg/kg was administered as a standard drug for comparison. Test compounds at two dose levels (100 and 200 mg/kg) were administered orally 30 min prior to administration of the writhing agent (0.6% v/v aqueous acetic acid, 10 ml/kg). The writhing produced in the animal was observed for 30 min and percentage protection was calculated for analgesic activity. The results are presented in Table 1.

**TABLE 1:** ANALGESIC ACTIVITY OF THE SYNTHESIZED COMPOUNDS

Compounds	Dose (mg/kg)	Mean±SEM	% Protection
1	100	34.33±1.616*	50.6
	200	23.00±1.252*	66.9
2	100	60.33±1.413*	13.1
	200	57.16±1.447*	17.7
3	100	38.66±1.781*	44.3
	200	27.83±1.839*	59.9
4	100	63.16±0.984*	9.1
	200	61.00±1.183*	12.1
5	100	66.5±2.152	4.3
	200	63.16±1.559*	9.1
Aspirin	100	9.06±0.808	86.1
Control (Acetic acid)	0.1 ml / 10 g	69.46	-

Each value expressed as Mean±SEM (n=6) in the acetic acid-induced writhing reflex model using student- t-test followed by one way ANOVA and

* P<0.05.

The analgesic activity was also determined by Eddy's hot plate method[20], using Swiss mice (n=6) of either sex selected by random sampling technique was used for the study. Pentazocine at the dose of 10 mg/kg (p.o.) was administered as standard drug for comparison. The test compounds at 2 dose levels (100 and 200 mg/kg) were administered orally. The temperature of the Eddy's hot plate was maintained at 55±0.5^°^. The animals for the experiment were chosen when the animal reaction (licking of the fore paws or jumping response) was within 10 s. After drug treatment every 30, 60, 90 and 120 min the reaction time was noted. The cutoff point of 30 s was observed to prevent the paw damage and data are presented in Table 2.

**TABLE 2:** ANALGESIC ACTIVITY OF THE SYNTHESIZED COMPOUNDS

Compounds	Dose (mg/kg)	Pain reaction time (min)				
		
		0	30	60	90	120
1	100	3.2±0.31	5.2±0.47*	11.3±0.58*	11.1±0.63*	10.8±0.49*
	200	3.4±0.36	6.4±0.53*	14.1 ±0.68*	14.3±0.72*	13.9±0.63*
2	100	2.8±0.28	3.5±0.31	4.0±0.98	3.8±0.38	3.5±0.42
	200	3.1±0.25	3.7±0.47	3.9±0.76	4.1±0.62	3.9±0.55
3	100	3.3±0.35	7.9±0.51*	9.4±0.54*	9.1±0.41*	8.7±0.47*
	200	3.4±0.37	8.7±0.57*	12.1 ±0.78*	11.5±0.71*	11.1±0.61*
4	100	2.7±0.42	3.1±0.29	4.0±0.94	3.8±0.41	3.4±0.53
	200	3.3±0.47	3.5±0.45	4.1±0.73	3.7±0.68	3.5±0.56
5	100	3.2±0.39	3.4±0.5	3.9±0.57	3.5±0.54	3.6±0.47
	200	3.5±0.49	3.3±0.48	4.2±0.63	3.3±0.57	3.4±0.52
Pentazocine	10	3.3±0.47	9.4±0.89*	12.1 ±0.62*	14.9±0.71*	14.6±0.59*

Each value expressed as Mean±SEM (n=6) in the Eddy's hot plate method using student- t-test followed by one way ANOVA and

* P<0.05 compared to 0 min reaction time

Antiinflammatory activity[21] was determined by carrageenan-induced paw edema method in Wistar rats (n=6) of either sex selected by random sampling technique. Indomethacin (20 mg/kg, p.o.) was administered as standard drug. The test compounds were administered at two dose levels (200 and 400 mg/kg) orally 30 min prior to the administration of carrageenan (0.1 ml) in the plantar region of the paw. The paw volumes were measured using plethysmograph at 1, 2, 3, 4 and 5 h after carrageenan administration. The results are presented in Table 3.

**TABLE 3:** ANTIINFLAMMATORY ACTIVITY OF THE SYNTHESIZED COMPOUNDS

Compound	Dose (mg/kg)	% Reduction of edema (h)				
		
		1	2	3	4	5
1	200	16.7±0.07	29.0±0.03	38.2±0.04*	21.2±0.06	18.2±0.03
	400	22.3±0.04	34.2±0.06*	47.3±0.07*	49.4±0.02*	43.4±0.03*
2	200	9.2±0.05	11.0±0.03	13.0±0.06	10.0±0.09	7.0±0.08
	400	12.1 ±0.08	15.0±0.03	21.0±0.06	23.0±0.01	20.0±0.07
3	200	19.9±0.05	28.2±0.09	45.6±0.07*	39.2±0.06*	21.3±0.02
	400	25.3±0.07	31.3±0.04*	52.3±0.09*	46.7±0.04*	28.4±0.08
4	200	9.2±0.07	13.1 ±0.04	15.2±0.03	16.3±0.09	14.7±0.04
	400	12.5±0.04	14.3±0.05	21.6±0.01	19.7±0.05	16.3±0.07
5	200	11.2±0.09	17.3±0.03	24.7±0.07	15.4±0.04	11.5±0.05
	400	17.2±0.05	21.4±0.07	26.4±0.08	28.1±0.02	20.5±0.07
Indomethacin	20	31.3±0.06	64.5±0.01	79.4±0.04	80.1±0.03	81.2±0.02

Each value expressed as Mean±SEM (n=6) in carrageenan-induced paw edema method using student- t-test followed by one way ANOVA and

* P<0.05

Antibacterial activity[22] of the test compounds (10 μg/disc) were tested against *B. subtilis, B. cereus, S. epidermidis, S. aureus K. pneumoniae* and *E. coil* using nutrient agar medium (Hi-Media Laboratories, India). The antifungal activity of the compounds was tested against *C. albicans* and *A. niger* using Sabouraud dextrose agar medium (Hi-Media Laboratories, India). The sterilized (autoclaved at 120^°^ for 30 min) medium (40-50^°^) was inoculated (1 ml/100 ml of medium) with the suspension (10^5^ cfu/ml) of the microorganism (matched of McFarland barium sulphated standard) and poured into a Petri dish to give a depth of 3-4 mm. The paper impregnated with the test compounds was placed on the solidified medium. The plates were pre-incubated for 1 h at room temperature and incubated at 37^°^ for 24 h and 48 h for antibacterial and antifungal activity respectively. Ciprofloxacin (10 μg/disc) and ketoconazole (10 μg/disc) was used as standard for antibacterial and antifungal activity, respectively. The observed zone of inhibition is presented in Table 4.

**TABLE 4:** ANTIMICROBIAL ACTIVITY OF THE SYNTHESIZED COMPOUNDS

Compounds	*In vitro* Activity Zone of inhibition in mm (MIC in μg/ml)								
	
	*S. aureus*	*S. epidermidis*	*B. cereus*	*B. subtilis*	*P. aureus*	*K. pneumonia*	*E. coli*	*A. niger*	*C. albicans*
1	21	14	21	19	20	19	16	19	23
	(5.5)	(6.5)	(4.5)	(5.5)	(4.5)	(6)	(7)	(5.5)	(4.5)
2	18	17	14	17	16	13	14	28	21
	(6.5)	(5.5)	(7)	(6.5)	(5.5)	(7.5)	(7.5)	(3.5)	(5.5)
3	20	18	20	18	22	21	19	21	22
	(5)	(4)	(5.5)	(5.5)	(3.5)	(4.5)	(5.5)	(6)	(5.5)
4	19	16	15	16	15	16	17	19	20
	(6)	(5)	(6.5)	(7.5)	(6)	(5.5)	(6.5)	(6.5)	(6)
5	15	13	19	14	17	18	13	22	21
	(7.5)	(7.5)	(6)	(8)	(7)	(5)	(7)	(6)	(4.5)
Ciprofloxacin (10 μg/disc)	29	22	28	27	26	25	30	-	-
Ketoconazole (10 μg/disc)	-	-	-	-	-	-	-	28	26

Minimum inhibitory concentration (MIC)[23] of the test compounds were determined by agar dilution method. A stock solution of the test compounds (10 μg/ml) in dimethylformamide was prepared and graded quantities of the test compounds were incorporated in specified quantity of molten sterile agar (nutrient agar for antibacterial activity and Sabouraud dextrose agar medium for antifungal activity). A specified quantity of the medium (40-50^°^) containing the compound was poured into a Petri dish to give a depth of 3-4 mm and allowed to solidify. The microorganisms were then streaked on agar plate and the plates were incubated at 37^°^ for 24 and 48 h for bacteria and fungi, respectively. The MIC was considered to be the lowest concentration of the test substance exhibiting no growth of bacteria or fungi. The observed MIC values are presented in Table 4.

All the spectral data was consistent with the assigned structure of the compounds. The ^1^H-NMR spectral data of 1 consisted of one singlet (imine proton, N=CH), two doublets (2'-H, 6'-H with J=9.1 Hz; 3'-H, 5'-H with J = 8.9 Hz), two triplets (morpholino protons with J=7.6 and J=8.1 Hz) and multiplet (phenyl protons) peaks. The spectral data of 2 consisted of two doublet (5”-H, with J=7.2 Hz; 3'-5'-H with 8.7 Hz) two triplet (morpholino protons with 7.8 Hz) and a multiplet (phenyl protons) peaks. The spectral data of 3 consisted of one singlet (2”-H), two doublets (3',5'-H with 8.9 Hz; 2',6'-H with 8.9 Hz) two triplet (morpholino protons with 7.7 Hz) and multiplet (phenyl protons) peaks. The spectral data of 4 consisted of a doublet, two triplet and multiplet (phenyl protons) peaks. The spectral data of 5 consisted of two doublets (-N=CH-CH with J=4.2 Hz, -CH=CH-ph, with J=6.0 Hz), triplets and multiplet (phenyl protons) peaks.

The ^13^C-NMR spectral data of all the compounds consistently exhibited peaks for the common functionalities present in the series of compounds such as C=N, morpholine and phenyl side chain carbons. The peak corresponding to C=N in 1, 2, 3, 4 and 5 was 154.3, 148.3, 159.9, 159.6 and 164.2, respectively. The peak corresponding to morpholino carbons representations in 1, 2, 3, 4 and 5 was 66.9, 50.3; 66.2, 50.8; 66.7, 50.5; 66.5, 50.7 and 66.1, 50.4, respectively. The peak corresponding to phenyl carbons (representative) in 1, 2, 3, 4 and 5 was 128.1, 128.6, 128.4, 127.9, and 128.7, respectively. The peak corresponding to C-Cl and C-NO_2_ was 134.8 and 147.7, respectively.

The mass spectral data revealed that all the compounds exhibited parent peak (M^+^) consistent with the assigned molecular formula. The fragmentation peaks correspond to the hypothesis of the fragmentation pattern of the compounds. The base peak of 1, 2, 3, 4 and 5 was 119, 169, 119, 139, and 131, respectively. The base peaks were found to be consistent with the proposed fragmentation hypothesis and according to the nature of the substituents.

N-benzylidine-2-morpholoinobenzenamine (1) and N-(3-nitrobenzylidine)-2-morpholino benzenamine (3) exhibited significant analgesic and antiinflammatory activities. Remaining compounds 2, 4, and 5 showed mild analgesic and antiinflammatory activities. All the synthesized compounds exhibited significant antibacterial and antifungal activity with an MIC range of 3.5-7.5 μg/ml. N-(3-nitrobenzylidine)-2-morpholino benzenamine (3) was found to exhibit the highest anti-microbial activity against *S. aureus* (5 μg/ml), *S. epidermidis* (4 μg/ml), *B. cereus* (5.5 μg/ml), *P. aeruginosa* (3.5 μg/ml), *K. pneumoniae* (4.5 μg/ml), *E. coli* (5.5 μg/ml) and *C. albicans* (5.5 μg/ml). Compound 2 exhibited highest activity against *A. niger* (3.5 μg/ml). The compounds were active against all the tested microorganism with a range of MIC values for *S. aureus* (5-7.5 μg/ml), *S. epidermis* (4-7.5 μg/ml), *B. cereus* (4.5-6.5 μg/ml), *B. substilis* (5.5-8.0 μg/ml), *P. aeruginosa* (3.5-7.0 μg/ml), *K. pneumoniae* (5-7.5 μg/ml), *E. coli* (5.5-7.5 μg/ml), *A. niger* (3.5-6.5 μg/ml) and *C. albicans* (4.5-6 μg/ml).

The results of the present study show that compound 1 containing unsubstituted phenyl ring in 2^nd^ position of the 4-phenyl morpholine exhibits greater activity than the substituted one. Out of the synthesized compounds the nitro substituted compound 3 exhibits more activity than the chloro substituted compound 4, which in turn exhibits more activity than furyl substituted compound 2. Compound 5 exhibits lesser activity.
